# “The male elephant in the room”: a qualitative evidence synthesis exploring male experiences of eating disorders

**DOI:** 10.1186/s40337-022-00614-w

**Published:** 2022-09-02

**Authors:** Emily Coopey, George Johnson

**Affiliations:** Centre for Applied Psychology, School of Psychology, University of Birmingham, Birmingham, UK

**Keywords:** Eating disorder, Male, Masculinity, Men, Meta-ethnography, Metasynthesis

## Abstract

**Background:**

Men are believed to be underrepresented in eating disorder services; there are many reasons presented to account for this such as a lack of recognition and detection. Due to the physical and psychological complexity of an eating disorder it is important to understand this underrepresentation. This qualitative evidence synthesis aimed to explore the literature relating to men’s experiences of an eating disorder, in order to synthesise the findings and offer a more coherent understanding.

**Method:**

A systematic search of the literature was undertaken. Inclusion and exclusion criteria were applied to the findings resulting in 14 papers deemed suitable for inclusion. A Meta-Ethnographic approach to synthesising the data of the 89 participants was undertaken.

**Results:**

Four themes were identified: ‘Societal Construction of the Perfect Male’; ‘Striving to Maintain a Masculine Identity’; ‘The Interconnectedness of Control and Self-Worth’, and ‘The Hidden Man’. There appeared to be an underlying concept relating to the conflict of being a man, with what is perceived to be a ‘woman’s illness’ and how this challenged the men’s experiences of masculinity.

**Conclusions:**

Being a man with an eating disorder conflicted with societal norms, exacerbating their experience of having an eating disorder.. To resolve this, gendered norms need to shift, at societal level as well as considering how best to improve understanding and recognition of men with an eating disorder at the first point of help seeking.

## Plain English Summary

Men are underrepresented in eating disorder (ED) services; are less likely to notice symptoms of an ED, and have this identified by professionals. To date, there has been limited research into the male experience of ED’s and this appears to be related to a difficulty with getting men involved in studies, as well as the lack of men in services. This review synthesised the existing literature, and four themes were generated: ‘Societal Construction of the Perfect Male’; ‘Striving to Maintain a Masculine Identity’; ‘The Interconnectedness of Control and Self-Worth’, and ‘The Hidden Man’. The results highlighted that the male experience is complex and influenced by societal assumptions and gender norms. EDs are predominately seen as a ‘women’s illness’, which affected the male experience in many ways but most importantly it prevented help seeking and recognition of eating disorders in men, arguably impacting their access to treatment. It is important to change the expectations we place upon men; enabling them to ask for help when needed in ways we allow women. Addressing societal pressures, combined with targeted training to improve understanding at the first point of seeking help, ensuring appropriate recognition of an ED and timely intervention for men.

## Background

### Eating disorders in men

Men are estimated to account for between 10 to 25% of the estimated 1.25 million eating disorder (ED) cases in the UK, which is believed to be an underrepresentation [1]. The underrepresentation is considered in the context of research which highlights the role of gender in help seeking, women are more likely to seek contact with a General Practitioner (GP) than men [2]. The stigma attached to mental health is likely to further impact this underrepresentation; research has highlighted that men are less likely to exhibit help seeking behaviours in relation to mental health support [3]. Further gender disparity is apparent, for example women are more likely to get a diagnosis of depression than men even when presenting with the same scores on a standardised measure [4]. Whilst there are apparent biological, genetic and personality trait differences between men and women, there is a wealth of evidence to indicate that these cannot always account for the differences in mental health diagnoses different genders receive [5].

There are numerous factors that are believed to contribute to the underreporting of men experiencing an ED. Research has highlighted that men tend to present with a less severe ED psychopathology [6], and this may account for the limited number of men reported to be experiencing an ED due to a lack of recognition by professionals. However, the role of societal norms may further limit the detection of EDs in men by professionals; as these are often under-reported and misunderstood [6, 7]. If there is a general view that men do not experience EDs, or that the male ED experience is rare or less severe, then it may inhibit professionals from asking questions or undertaking appropriate investigations instead attributing ED symptomology to another cause [8]. Furthermore, there is a difference in the way men and women present as men may be striving to attain muscle mass [7], which may be viewed as normal and acceptable, hindering recognition of an ED. Modern Western culture floods men with messages about their appearance and body [9]; and there is extensive research highlighting how men feel the need to conform to the mesomorphic ideal [10–13]. It is believed that in Western societies men strive for defined muscle and low body fat, often following strict rules in order to achieve this [14] and as such their behaviours may be vastly different to those of a woman with an ED [15].

Furthermore, there appears to be an expectation that men ‘man up’ and manage their vulnerabilities [16, 17]. Although women also experience  societal pressures, it has been argued that as these have been recognised over a long period of time, they have become more adept at challenging these societal ideals [18], whereas when men feel pressure to conform to perceived ideals they have fewer resources to deal with this. Indeed, it has been argued that social expectations continually reinforce the notion that men do not talk about their emotions, or body dissatisfaction [18] leading to secrecy and an avoidance of challenging norms, including through treatment-seeking.

Despite the difference in presentation, the lack of recognition and the barriers men experience with regards to help seeking, it is believed that men do not have a poorer prognosis than women with regards to treatment [7]. However, the underreporting of men with an ED [7, 19] highlights the need for further research in order to more clearly understand their experience. To date there are two qualitative reviews exploring men’s experiences of treatment for ED in 2018 and 2014 (54, 208) [6, 20]. It is important to review and synthesise the currently available research in order to have the clearest contemporary sense of men’s experience of an ED.

## Method

### Aims of the review

The review aimed to synthesise the existing literature regarding men’s experiences of an ED to enable a broader understanding of an underrepresented population.

### Type of review

A Meta-Ethnographic approach to synthesising the literature was undertaken, informed by Noblit and Hare [21] utilising data to enable both first and second order constructs [22]. Noblit and Hare’s [21] seven phases of Meta-Ethnography are outlined in Table 1. Meta-Ethnography enables the rigorous development of a literature review through the comparison and analysis of a data set, arriving at new interpretations [21].

**Table 1:** Outlining the seven phases of Meta-Ethnography [21]

Phase	Description
1. Getting started	Identify an area of interest whilst considering if a synthesis of the topic is required
2. Deciding what is relevant	Selecting studies for inclusion in the synthesis. Making decisions regarding inclusion, exclusion and assessing quality
3. Reading the studies	Repeated reading of the studies whilst extracting key concepts
4. Determining how the studies are related	Exploring the relationship between the extracted key concepts to enable an understanding of how the studies are interconnected
5. Translating the studies into one another	Exploring the key concepts across all studies, looking for presence or absence of key concepts
6. Synthesising translations	Creating concepts across studies. Exploring the relationship between studies and deciding if synthesis is refutational, reciprocal or line of argument
7. Expressing the synthesis	Compiling the synthesis and delivering it to the intended audience.

Further, this approach enables the development of conceptual understandings of individual experience, even in areas of established research [23]. Meta-Ethnography is believed to usefully enable the synthesising of an individual’s experience of an illness [24] through a robust method that enables the development of interpretations across the studies reviewed [25], thus a Meta-Ethnographic approach was of particular relevance in the under-researched area of men’s experiences of an ED.

### Phase 1. getting started

Exploratory reviews of the literature for Phase one identified only one systematic review focused on men’s experiences of treatment [20], which included four papers, therefore exploration of research developments in the past five years was undertaken. However, further searches of the literature highlighted there was not sufficient research regarding men’s experiences of treatment, and so the focus of the review expanded to explore men’s experiences of having an ED more broadly.

### Phase 2: deciding what is relevant

#### Systematic literature search

On developing a research question, phase two of Noblit and Hare’s [21] approach was implemented by undertaking a systematic search of the literature.

#### Search strategy

The search strategy was developed (see Table 2), and applied to a number of databases. The only variation to this search strategy was for the database Web of Science where the search term ‘NOT rats’ was included to reduce the number of results relating to animal studies. ‘Grey literature’, which for the purpose of this study was unpublished (examined) theses, provided additional research studies.

**Table 2:** Database search terms

Search	Description
1	“Eating Disorder*”
2	Male* OR Men
3	Experience OR “Lived Experience”
4	1 AND 2 AND 3
5	LIMIT 5 to “Qualitative (best balance of sensitivity and specificity)”

*Indicates a truncation

#### Inclusion/exclusion criteria

A number of inclusion and exclusion criteria were applied to the literature identified during the systematic search strategy as outlined below in Table 3. A time frame was not applied to the papers because of the sparsity of research in the area.

**Table 3:** Inclusion and exclusion criteria applied to identified literature

Inclusion criteria	Exclusion criteria
Participants were men	Not focused on EDs
Participants over the age of 18	Not qualitative
Participants had an experience of an ED that was defined by a diagnosis	Not focused on men’s experiences
Qualitative research exploring experiences	Not clear if participants were men
English language	Experience of someone other than the person experiencing an ED
	Literature reviews including systematic reviews and meta syntheses
	No clear analytic technique allowing for comparison of thematic structures

One study included two 17-year-old male participants but was included as the majority of participants were over the age of 18 and the paper contributed effectively to answering the question proposed by the review. This review focused on adult males both to ensure homogeneity and because there is very limited research available with adolescent males.

#### Systematic screening process

**Fig. 1:**
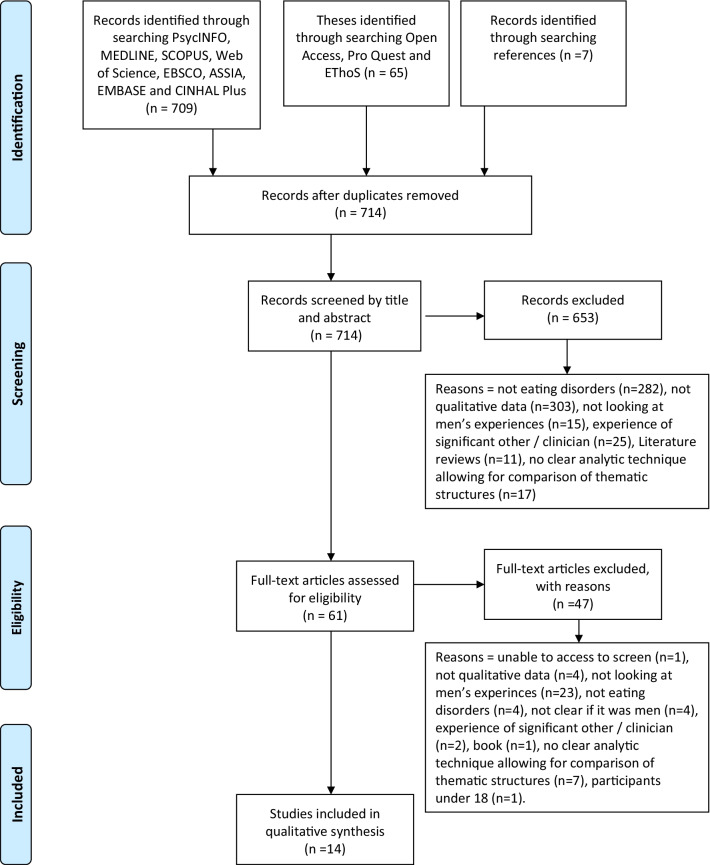
PRISMA diagram displaying systematic screening process [26]

The systematic screening process resulted in 14 papers to be included in the synthesis via the process of Meta-Ethnography (see Fig. 1). Of these six were from the ‘grey literature’. An overview of the 14 papers is provided in Table 4.

**Table 4:** Overview of the articles to be included

Author(s), year of publication, country	Study title	Key research question/aim(s)	Participants	Data collection and analysis
Oickle, 1998 [27] Canada	A needs assessment: resources for males with EDs	What is the nature of the experiences with available resources or males with EDs and associated health care professionals?	Eight men, aged 18–42, diagnosed with an ED	Interviews Inductive analysis
Drummond, 1999 [28] Australia	Life as a male 'Anorexic'	To highlight the significance of EDs amongst men	Eight men, aged 18–42, all had contact with SAABNA for ED presentation	Interviews Inductive approach
Drummond, 2002 [29] Australia	Men, body image, and EDs	To explore issues relating to masculinity and men's health with respect to eating-disordered men	Eight men, aged 18–42, all had contact with SAABNA for ED presentation	Interviews Inductive approach
Ashuk, 2004 [30] Canada	Narratives of males with EDs	What is the nature of the experiences of males who have an ED?	Two men over 18, diagnosed with an ED and accessing ED services	Interviews Narrative research
Wertheimer, 2006 [31] America	Gay men with eds and food, body image and exercise concerns: a group treatment approach	Explore the participants’ experiences in the group and the features of the group that may have contributed to its impact	10 men, aged 30–47, who met diagnostic criteria for an ED. Eight were Caucasian, one mixed race and one Mexican American	Interviews Grounded theory
Robinson, Mountford, & Sperlinger, 2012 [32] United Kingdom	Being men with EDs: perspectives of male ED service-users	What is it like for men to live with an ED? What is it like for men to seek treatment for an ED? What is it like for men to receive treatment for an ED?	Eight men, aged 24–56, all with a diagnosed ED and receiving treatment for the ED. All participants were white British or white Irish	Interviews IPA
Björk, Wallin, & Petterson, 2012 [33] Norway & Sweden	Male experiences of life after recovery from an ED	To explore adult males experience of recovery from an ED	15 Men, aged 19–52, with a previous diagnosed ED	Interviews QPA
Markham, 2013 [34] United Kingdom	Exploring men’s accounts of understanding and seeking help for problems with eating	To explore how men (in the UK) made sense of the development of an ‘ED’ and their experiences of living with and seeking help for the ‘ED’	Eight men, aged 22–53, involved with an ED Charity with self-identified ED. All participants were white British	Interviews IPA
Räisänen & Hunt, 2014 [35] United Kingdom	The role of gendered constructions of EDs in delayed help-seeking in men: a qualitative interview study	How do men make sense of their early (and later) signs and symptoms of disordered eating? How do they realise something might be wrong and require intervention? Are there perceived barriers to accessing primary care (or other) services for men with EDs? What are men’s experiences of health professionals’ responses to their initial presentations of ED signs and symptoms?	10 Men, aged 17–25, eight diagnosed with an ED and two self-identified. Eight were white British, one was Latino and one was mixed race. Six were students, two were employed and two were unemployed	Interviews IPA
Spyrou, 2014 [36] United Kingdom	Exploring men’s experiences and understanding of binge ED: an interpretative phenomenological analysis	How do men experience and understand BED? How do men with BED experience and understand the process of seeking, accessing and receiving treatment(s)	Six men, aged 22–50, with a formal diagnosis of (BED). Four participants were employed full time and two were self-employed	Interviews IPA
Wallin, Pettersen, Björk, & Råstam, 2014 [37] Norway & Sweden	A qualitative study of males’ perceptions about causes of ED	How former male patients perceived causes of onset of their ED	15 Men, aged 19–52, who had been diagnosed with an ED. Had received treatment and deemed to be in recovery	Interviews QPA
Pettersen, Wallin, & Björk, 2016 [38] Norway & Sweden	How do males recover from EDs? An interview study	To investigate what males experience as helpful in their recovery process from an ED	15 Men, aged 19–52, with a formal diagnosis of an ED. Deemed to have completed treatment	Interviews Content analysis
Thapliyal, Mitchison, & Hay, 2017 [39] Australia	Insights into the experiences of treatment for an ED in men: a qualitative study of autobiographies	To explore the experiences of men who ever had any form of treatment for an ED	Six men, aged 25–50, with a diagnosis of an ED. Various stages of recovery. The occupations of the authors were: Restaurant critic, Salesperson, Writer and Producer, Library staff, Consultant, Writer & Speaker & Professional ED Counsellor	Autobiographical accounts Thematic analysis
Tresca, 2018 [40] United Kingdom	An exploration of men’s experiences of motivation to change in relation to their journey with Anorexia Nervosa	Participants’ experiences of Motivation to Change including but not limited to what drives, impedes and challenges their journey with AN	Eight men aged 20–44, defined by researcher to meet criteria for an ED. Six were classed as recovered and two were in recovery. Four participants reported their nationality as British, one reported UK, one reported USA, one reported Italian and one reported Canadian. Seven men were employed and one was a full-time student	Interviews IPA

A total of 89 participants are included in the review. Some of the studies used the same data set [28, 29, and 33, 37, 38]. Where participant information on race, ethnicity, gender identification and socioeconomic status was available it was included in Table 4. All participants were either diagnosed with an ED or believed to meet the diagnostic criteria by the researchers. The age range of participants was predominantly 18–65 and the majority of studies were conducted in the United Kingdom. The predominate approach used by the studies was Interpretative Phenomenological Analysis (IPA) but a variety of approaches were used.

#### Quality appraisal

The quality of the studies included was appraised using the National Institute for Health and Care Excellence (NICE) recommended Methodology Checklist for qualitative studies [41]. The checklist was applied systematically to each study.

Ten studies were deemed to be of good quality in that they met most or all of the checklist criteria. Two studies [35, 38] were deemed to be of a fair quality and two papers [28, 29] were deemed to be of poor quality. However, due to the lack of research in this area, these papers were judged to contribute to the review and were therefore included. For the majority of studies assessed, it was a lack of reporting of information that contributed to a reduction in their scores on the quality criteria checklist, such as details around ethics and the role of the researcher.

To enhance validity a sample of the quality criteria ratings were compared with the research supervisor’s ratings and a sample were rated by a second rater. Any inconsistencies in the ratings were discussed until agreement was reached.

### Phase 3: reading the studies

Phase three focused on reading of the studies to enable the researchers to begin extracting relevant data. Noblit and Hare [21] recommend repeated reading of the studies at Phase three to enable the extraction of key concepts.

### Phases 4, 5 and 6: data analysis and synthesis

The final phases to analysing and synthesising the datah are phase 4: determining how the studies are related, phase 5: translating the studies, and phase 6: synthesising the translations. Whilst these are presented as independent phases, in practice the researchers’ moved between phases to enable synthesis and the creation of themes.

Once the key concepts and associated data had been extracted the researchers were able to explore the interconnectedness of the studies. Discussions were held throughout with research supervisors and peers to enhance the validity of the process. Triangulation was implemented in a variety of forms; regular fortnightly workshops and discussions with peers and supervisors, until mutual understanding was reached.

## Results

**Fig. 2:**
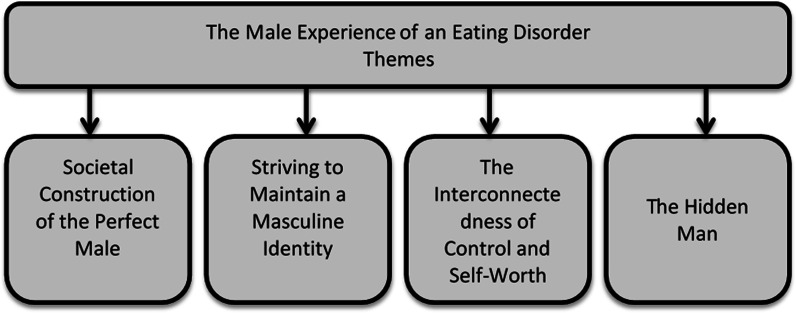
An overview of the themes

Four meta themes were created (see Fig. 2), each theme encapsulated elements of the experience of men living with an ED.

### Societal construction of the perfect male

The importance of the impact of societal norms on the men was noted. Oickle ([27]; pp. 136), stated: “Participants expressed feeling the need to live up to an unbreakable image for men set by society.” and this idea was supported by a number of papers [27, 29, 30, 34–37, 39]. It appeared that the men were acutely aware of an ideal, as one participant described the: “…ideal male physique…combines thinness and muscularity, which they associate with a masculine stereotype of being a man” ([36]; pp 62). Yet participants also felt that their appearance contrasted with this as a participant explained: “I don't fit the cultural model of masculinity…” ([29]; pp 8). Furthermore, certain characteristics were deemed important for men by society, as Oickle highlighted: “…that nothing can hurt men, that they are not emotional, and that they can handle all problems without help” ([27]; pp 136), reinforcing this notion of a perfect man: “…which is impossible for some of us to have.” as reported by a participant ([29]; pp 9). The idea that men should not need to seek help appeared to be reinforced by societal constructs as Oickle ([27]; pp 138) highlighted: “The ‘strong man’ image was expressed as one of the reasons men may not seek treatment or information to help them deal with their ED”. One participant highlighted the detrimental impact of this as “Guys would suffer longer because they won’t come forward, they won’t seek therapy because of the stigma” ([36]; pp 69).

Ashuk ([30]; p 95), highlighted the role of the media in overemphasising: “…physical strength, force, aggressiveness, competitiveness and independence in males.”, with participants noting the reinforcement of the pressure relating to  societal norms  by the media [30, 34]. Furthermore, there was a sense that although the men struggled to fit with these norms this should not impact upon them, yet the pressure to conform was seen as a contributing factor to the development of their ED [29, 37]. Whilst others highlighted gendered norms influenced a lack of internal [35] and external [34] recognition of the ED, as supported by Räisänen, & Hunt: “Rather than referring him for psychological treatment he was, in his words, told to ‘man up’ and ‘not be weak but be strong and deal with the problem’” ([35]; pp 5).

Societal expectations for men appeared to create an impossible position for the study participants whereby they experienced an external pressure to act and behave in a certain way yet: “…admitting to low self-esteem and distorted body image goes against the expectations of what men are supposed to feel and think about” ([27]; pp 139). Markham ([34]; pp 43) highlighted that men experience a: “…paradox of an increased pressure to conform to a certain image of toughness and strength, yet it being considered unacceptable for men to talk about, or express any form of concerns around, diet or body image…”. This impossible situation seems to maintain the ‘stuckness’ of men who experienced something that does not fit within the societal constructs of being a man, which was further supported by one participant: “I don't fit the cultural model of masculinity because I'm not very muscular” ([29]; pp 8).

### Striving to maintain a masculine identity

A key concept emerged relating to the experience of being a man and trying to maintain masculine ideals whilst admitting to needing help. Drummond highlighted participants believed to be flawed: “I get worried that they might now see me as less of a man. Flawed. Not as strong”, as reported by one participant ([29]; pp 8) and many papers contributed to the idea that men did not want to be seen as flawed [29, 32, 34, 36, 37]. There was a sense that accepting they had an ED impacted on how they perceived themselves, as one participant explained: “well, the disclosure, the shame of having a problem” ([27]; pp 113). There was a sense that being flawed related to wider societal constructs as highlighted by Markham ([34]; pp 38): “…expectations of what it meant to be a *‘boy’s boy’* and the participant’s positioning in the boyhood network.”

Moreover, striving to maintain a masculine identity appeared to be situated within a sense that the men felt they did not fit in, and therefore, maintaining a perception of a masculine identity was important [27, 29, 34, 36, 37]. These papers contributed to the concept that men worried about a sense of de-masculinisation. Robinson, Mountford, and Sperlinger ([27]; pp 181), highlighted that: “Many felt there was something unacceptable about them which had to be compensated for…”. The notion that participants needed to live up to masculine stereotypes was highlighted as the men shared a sense that they felt a burden to live up to expectations, yet experiencing an ED meant they could not live up to ideals which ultimately reinforced the notion that they did not fit in, as one participant described: “My ideal body for a masculine guy is a white T-shirt that fits and a pair of blue jeans that fit” ([29]; pp 8). The very thing that they felt might have helped them achieve a masculine ideal in terms of physical attributes was now the very thing preventing them from connecting with their sense of masculinity.

Furthermore, it was felt that the masculine ideals placed upon men referred to the notion that men should not be seen as anything but resilient: “I don’t want to be seen as a weak… it’s a symptom of not being able to cope” reported a participant ([32], pp 181), several papers contributed to this idea [27, 28, 37]. Markham ([34]; pp 48) highlighted that one participant spoke: “…of his ‘need’ to carry out certain activities…” indicating the: “…pressure he feels to perform as a man.”, such as: “…consistently earn a wage…”. Further reinforcing the conflicting experience of having an ED and upholding masculine roles and ideals. Spyrou [36] focused on men’s experiences of BED and presented the notion that binge eating could be seen as more acceptable for men as eating large quantities of food is often seen as manly and it is widely accepted that men eat more than women.

### The interconnectedness of control and self-worth

Participants appeared to need a sense of control, which fitted within a wider sense of internal personal standards [28–33, 37, 38, 40]. This theme appeared to largely embody an internal standard that participants were aiming for, yet there was an element of achieving this internal standard for external recognition [28, 30, 34, 37]. It was felt that achieving internal and external standards enabled the participant to achieve a sense of self and self-worth.

Ashuk ([30]; pp 97) highlighted that participants were required to maintain a: “…discipline [that] required determination and fortitude…”, and Drummond ([29]; pp 23) indicated the men knew: “…how to successfully compete within the context of their own personal illness.” ,reinforcing the notion that participants were aiming to achieve an internal standard that helped shape their identity and self-worth. The men were able to improve low self-esteem by achieving the high standards they set themselves, but this in turn seemed to fuel the internal standards set [37].

Striving for perfection in the form of an ED provided an additional benefit in the form of disconnect with their emotions, as Wertheimer [31] reported the ED enabled: “a way to cope with or disconnect from difficult emotions” (pp 176), creating a sense of numbness as one participant described: “if I’m not doing it, I have to feel my feelings, which is difficult” (pp 176), highlighting the perceived advantage of an ED. Feelings such as: “disdain and disgust were often levelled at their personal appearance, heavily impacting on individual masculine identity” ([38], pp 8). The dislike of oneself further reinforces the notion that achieving internal standards for external validation may enhance ones sense of self and is therefore a way of measuring self-worth.

It was felt that maintaining control was central to the ED and this appeared in a number of papers: “…controlling the amount of food he put in his mouth was important in being successful at what he does as a man” ([28]; pp 84). This was reinforced by the idea that the men were relinquishing control in order to recover [28, 39] and by recovering Ashuk ([30]; pp 102) indicated: “…to ask for help, would have been to admit failure.” The notion of conflict between having an ED or recovering further reinforced the ambivalence the men felt in relinquishing control as there was a duel: “…desire to “fix” their bodies and other perceived flaws and the desire to accept themselves as they were.” in order to recover ([31]; pp 199). It is questioned if the idea of maintaining control was central to the men’s experiences yet as they entered recovery was there a realisation that they were never in control; they were controlled by the ED.

A smaller number of papers [28, 30, 34, 37] contributed to the notion that participants’ internal standards were driven by their need for external validation. Markham ([34]; pp 66) highlighted participants: “…felt the need to achieve and be good at something… he needed to be successful himself to affect others’ perception of him…”. Despite the internal drive to succeed there remained an element of needing validation from others as one participant reflected: “Being perfect meant being a better person. I wanted for others to see me as perfect” ([30]; pp 90), which ultimately led to a desire to not fail. It is questioned if the men felt that achieving an internally set standard within an ED enabled them to feel as if they were successful. Men appeared to use comparison to measure their success; with one participant claiming to feel a sense of achievement as: “…no-one trains as hard…” ([28]; pp 85) whilst another highlighted the comparison drove the desire to achieve [37], indicating the need for external validation. Obtaining external validation appeared to fit with the notion of creating a sense of self and measuring self-worth, yet it remained interconnected with internal standards as achieving internal standards enabled external validation which appeared to impact on the individuals’ sense of self. Self-worth is believed to be determined by internal and external perceptions of an individual’s ability and linked with success [42]. It is further questioned how an experience of an ED impacts on a man’s sense of self and how this infiltrates the need to be in control and succeed

### The hidden man

The theme of men being ‘hidden’ developed from the literature [27–32, 34, 35]. It became apparent that the feelings associated with men wanting to hide centred on shame, which connected to a sense of feared stigma. It was felt that participants carried the ED as a: “…burdensome, shameful secret…” ([31]; pp 189) and a: “Lack of communication and feelings of isolation were noted by the men as prominent difficulties” ([27]; pp 141), reinforcing the drive for the ED to remain hidden. Robinson et al. ([32]; pp 181) highlighted that fear of how others would perceive them acted as a barrier for help seeking in the men, as: “ Some had told people about the ED and were met with disbelief and rejection”. The men were concerned about how they would be perceived by both peers and professionals, Ashuk highlighted how the men may have felt: “fear that they will be unfairly stigmatized, either from society or from their peers ([30]; pp 103). Feelings of isolation impacted on help seeking for the men, not only preventing them from accessing help but their knowledge and awareness of available help [27].

It was felt that the men remained hidden, as they believed they were the only one: “…alone as a man with an ED, as if they were the only ones” ([32]; pp 180). One participant remained hidden as he was not sure a man could have an ED: “I wasn’t even sure that men got it... There were no role models... so maybe then this is an abnormal thing...maybe this isn’t what I’ve got” ([34]; pp 45), reinforcing the almost impossible position of the men in understanding what was going on and obtaining help, making it all too easy for the ED to remain secret. Yet: “…learning that they were not alone in their concerns about food, body image, and exercise” ([30]; pp 183) proved to be important in enabling steps towards recovery. Furthermore, Wertheimer [31] highlighted the importance of reducing feelings of isolation during the process of recovery for men, indicating isolation is a maintaining factor for men experiencing an ED. This idea was supported by Oickle, who reported: “Feeling ‘alienated’, ‘isolated’, ‘alone’, ‘separated’, and like ‘the only one’” were noted as reasons for not accessing treatment and information resources” ([27]; pp 142). EDs in men appeared to remain hidden due to a shrinking focus, leaving the individual with not much but the ED [29] thus enabling the individual to keep their behaviours and psychopathology a secret. The very cyclical nature of being a man who keeps his ED hidden and therefore remains hidden is further impacted by societal expectations and normative behaviour as highlighted by a participant who described the challenge of being: “…open about [the ED] when you are not getting messages that it’s ok to be open about it” ([35]; pp 47).

There appeared to be a wider, overarching contributing factor to men remaining hidden in the context of societal norms and how these influence external individuals. It appeared that others did not consider the men to have an ED nor they did not consider it to be serious [29, 30, 35]; this resonated with friends and family [34, 35] and professionals reinforced the idea that only women get an ED [32]. Research highlighted: “When professionals are not recognizing an ED, the men…get better at "hiding" their condition” ([27]; pp 129). There was a sense that men were assessed in terms of their physical presentation as opposed to their psychological distress [29, 35], as highlighted by a participant: “you're not at the critical level” ([29]; pp 5), which is in keeping with normative experiences of accessing primary care with an ED [43, 44]. Furthermore, societal norms relating to men and men’s behaviour impacted on the men’s motivation to seek help [27], with men fearing how they would be perceived [32] and if they would be viewed as weak [29]. Further to a lack of recognition, this also manifested as missed or incorrect diagnosis [35].

## Discussion

This qualitative evidence synthesis highlighted a number of themes identifying the challenging experience of being a man with an ED. Men’s experience appears to be compounded by societal constructions and either an internal or external conflict in relation to societal expectations.

### Societal assumptions

The research analysed indicated that men’s experience of an ED occurred in the context of societal assumptions, which contributed to the illness remaining hidden. Throughout there was an overarching sense that men’s experience existed in, and could not be separated from, societal expectations of what it means to be a man and how this contributed to the hidden nature of men’s experiences of an ED. The role of societal norms in men’s experiences of an ED are complex and multifaceted; the theme of ‘Societal Construction of the Perfect Male’ highlighted how men were encouraged to strive to look a certain way [7, 29, 36] yet when they experienced an ED in the context of striving, their presentation was at odds with what society stipulated about men’s behaviours and emotions [18, 27, 34], which was reinforced by the theme ‘Striving to Maintain a Masculine Identity’. Conversely, whilst EDs remain under reported in both genders [7, 19], female illness behaviours are celebrated and encouraged by society and their body dissatisfaction normalised, whilst for men there is an expectation that they do not have body dissatisfaction, they do not diet and most importantly they do not discuss this with others [18]. An ED is wider than body dissatisfaction; it can be a means of managing difficult and unwanted emotions [6, 45, 46] yet the synthesis highlighted that men are not expected, and subsequently not encouraged, to discuss their emotional and mental wellbeing. The notion of not discussing emotions and mental health was highlighted in the theme ‘Striving to Maintain Masculine Identity’ as the men did not wish to be seen as weak or unable to cope.

Societal norms enable behaviours to remain hidden if they do not fit with a gendered stereotype, which was highlighted throughout the theme of ‘The Hidden Man’ as it emphasised the diagnostic difficultly encountered by men with an ED [35]. The men described being acutely aware of societal norms highlighted in the theme ‘Societal Construction of the Perfect Male’ and the challenges faced when not fitting with gender based behaviours in ‘The Hidden Man’. Societal norms leading to a lack of acknowledgement around men’s experiences of EDs enables the experience, and the men, to remain hidden; denying their individualised choice and action. When considering how widely distributed and entrenched societal norms are, it becomes apparent the battle men have to firstly understand their own experience as having an ED [32, 34] and secondly to seek help [30, 32]. The impact of societal constructions of men and what men expect of themselves needs to be considered in light of help seeking and how this may impact on men. If as a society we do not accept men as experiencing an ED then this will further impact on our understanding of men’s process of help seeking, treatment and recovery.

### Gendered norms

An overarching concept from the qualitative evidence synthesis was the experience of being a man with a woman’s illness; the notion of an ED being a woman’s illness and gendered norms further compounded the experience of the men. Whilst the impact of ‘gendered norms’ is interconnected with ‘societal assumptions’, this overarching concept was situated in the conflict the men experienced when believing to be engaging in behaviours that are normative for women. It was felt that the ED provided men with a maladaptive means of achieving control [29, 30] and addressing self-worth [37], as highlighted in the theme ‘The Interconnectedness of Control and Self-Worth’. Yet the overarching sense of the difficulties encountered by the men related to their experience of a ‘woman’s illness’. When attempts to maintain a sense of masculinity were made through ED symptomology, they were confounded by the awareness that the behaviours were stereotypically female [34]. The notion of women gaining a sense of control from an ED is supported by the literature [46] highlighting there is not a disparity between genders in this particular experience.

The experience of men having a ‘women’s illness’ was further intensified when considering the external pressure experienced by men to behave in line with gendered norms [18, 27, 35], as discussed in the theme ‘Striving to Maintain a Masculine Identity’. As the men struggle to live up to a gendered norm, they engage in behaviours associated with a women’s illness yet their gender roles reinforce that they should not engage in those behaviours, let alone talk about those behaviours, enabling the men’s experience of an ED to remain invalidated. Wertheimer [31] highlighted the ED enabled a disconnect from emotions, further highlighting the functionality for men as they are stuck between striving to achieve a sense of self as a man whilst being mandated by society to cope [17], it is possible the ED enabled them to maintain gendered norms as they were cut off from their emotions, presenting as stoic as expected of men. It is questioned how different the men’s experience of an ED would be if it was not situated within the parameters of what is perceived as a women’s illness; if men experiencing an ED were not caught in a paradox of conforming to gender norms as highlighted in the research regarding help seeking [2, 3, 5]. Markham [34] described the men needing to belong to a male network, presenting the idea that they are caught in a paradox of conforming to gendered norms and societal expectations regarding appearance, yet it is deemed unacceptable for men to discuss their body image. This notion that men should not talk about body image is present, so how do men make sense of engaging in ED behaviours to achieve a desired body image: “…you are not allowed to be concerned… there is a big male elephant in the room” ([34], pp 43).

### Comparison to other reviews of the literature

This qualitative evidence synthesis aimed to review men’s experiences of an ED as there is limited existing research exploring men’s experience and the synthesising of existing literature aimed to provide a greater understanding. The findings can be considered in light of the existing literature relating to men’s experiences of treatment and the literature exploring women’s experiences. Women’s experiences of recovery highlighted similarities to that of men as the women described the ED becoming part of their identity and lacking a sense of self [47] and developing a sense of identity as part of their recovery [48]. Duncan et al. [48] highlighted women experienced a loss of control with the increase in ED symptomology and many papers highlighted the need for the men to maintain control [26, 32, 39, 40] and the relinquishing of control related to recovery [27, 29, 38]. Thapliyal et al. [6] explored gendered experiences of treatment for an ED, highlighting how treatments for EDs challenged men’s identity, which is in keeping with the findings of this Meta-Ethnographic approach to synthesising data; men’s’ experiences of an ED impacted on their sense of self and masculinity.

### Critique of the review

A qualitative evidence synthesis is subjective in that it relies on interpretations, not only of the researcher synthesising, but also those of the original authors of the studies included in the review. To minimise the subjectivity of this Meta-Ethnographic approach to synthesising, triangulation was implemented in a variety of forms; regular workshops and discussions with peers and supervisors, until a mutual understanding was reached.

There were a small number of studies included in the qualitative evidence synthesis. However, the majority of the papers were deemed to be of good quality when they were appraised, although some of the studies were focused on a particular diagnosis or aspect of the treatment pathway, which limited the contribution they could make to the wider research question.

Whilst qualitative evidence syntheses enable qualitative studies to be drawn together to enable broader interpretations to be made, the generalisation of the interpretations made remain limited due to the small sample.

### Clinical implications

This qualitative evidence synthesis highlights the importance of societal assumptions and gendered norms in the understanding and treatment of men with an experience of an ED. In particular the theme ‘The Hidden Man’ highlights that men have an experience of not being heard or not being understood when help seeking. Research has highlighted a low incidence of ED cases presenting to GP services [49], and the need for training and better liaison between GP services and specialist ED services [49]. Improving understanding of EDs, through training and dissemination of information, at the first help seeking experience could positively impact the cyclic nature, and possibly address the fact men are still largely unaccounted for in ED services [50].

Whilst there is a recognition of the need to challenge societal pressures regarding women’s appearance [18] and an understanding of the impact this has on a women’s sense of self and worth [51, 52], as a society it has been argued that we are yet to realise the impact societal pressures have upon men and their sense of self-worth, despite western cultures appearing to overwhelm men with appearance related ideals [9]. Further work is needed to challenge gendered societal norms as currently there does not appear to be a drive to change the way men are portrayed in society. Societal norms would assume that men are not affected by body ideals and the messages portrayed in society [18], therefore their sense of self and worth is not affected.

Further research into men’s experiences of an ED is warranted to enable the synthesising of treatment experiences and diagnosis specific experiences. Recruitment of men to ED research has been historically problematic [53], which is understandable in the context of societal norms and gendered norms. However, to enable further research into men’s experiences of an ED, and therefore enhance understanding, the problems with recruitment need to be considered and addressed.

## Conclusions

This review highlighted the importance of understanding men’s experiences of an ED, and it is clear that there are challenges situated in societal norms and gendered expectations. These challenges prevented understanding on many levels: that of the men themselves; health care professionals, and society more widely, which evidently has implications for detection and treatment of ED’s in men. There was an ongoing conflict centered around what it meant to be a man whilst experiencing an illness associated with women. Finally it is apparent that there is a need to address existing and potentially harmful narratives, in order to enhance both the awareness and understanding of men’s experiences of an ED.

## Data Availability

The datasets used and/or analysed during the current study are available from the corresponding author on reasonable request.

## Abbreviations

ED: Eating disorder
GP: General practitioner
SAABNA: South Australia Anorexia Bulimia Nervosa Association
BED: Binge eating disorder
IPA: Interpretative phenomenological analysis
QPA: Qualitative phenomenographic approach
NICE: National Institute for Health and Care Excellence
